# Asymptomatic Peripheral Arterial Disease Among Jordanian Patients With Diabetic Foot Ulcer

**DOI:** 10.7759/cureus.73722

**Published:** 2024-11-15

**Authors:** Waqas Abu-Jableh, Ahmad Younis, Anees Hjazeen, Eman Mashaqbeh, Ehab Al-Sharif

**Affiliations:** 1 Advanced Diabetic Foot Nurse Specialization, Royal Medical Services, Amman, JOR; 2 Community Health Nursing and Biostatistics, Royal Medical Services, Amman, JOR; 3 RN-Wound Care Unit, Royal Medical Services, Amman, JOR

**Keywords:** asymptomatic, claudication, diabetes mellitus, peripheral arterial disease, toe brachial index

## Abstract

Background and objective

Peripheral arterial disease (PAD) is a common disorder that is usually associated with leg symptoms such as intermittent claudication (IC), which could be masked by peripheral neuropathy in diabetic patients. In this study, we aimed to assess the prevalence and associated risk factors of asymptomatic PAD among Jordanian patients with diabetic foot ulcers.

Methods

A cross-sectional study involving diabetic foot patients attending the diabetic foot clinic at the Medical City in Royal Medical Services in Amman, Jordan, was conducted from January 2023 to March 2024. The criteria for the diagnosis of asymptomatic PAD were as follows: patients with toe brachial index (TBI) <0.7 and no leg symptoms as determined by the San Diego Claudication Questionnaire (SDCQ).

Results

PAD was observed in 38 out of 133 patients (28.6%). Of these, 26 patients (68.4%) had asymptomatic PAD. Statistically significant associations were observed between PAD and male gender, former smoking, cerebrovascular disease (CVD), age, duration of diabetes, and high-density lipoprotein (HDL). Multiple binary logistic regression showed that ex-smoking and age had a significant impact on developing PAD; ex-smoker patients were 4.75 times [95% confidence interval (CI): 1.26-17.90, p=0.021] more likely to have PAD than non-smokers, and the odds ratio (OR) of having PAD increased by 1.05 times for each additional year of patient age (95% CI: 1.004-1.095, p=0.032). In investigating factors associated with asymptomatic PAD, only hypertension showed significant results.

Conclusions

The prevalence of asymptomatic PAD is relatively high in diabetic foot patients. PAD may delay wound healing and lead to limp loss and poorer quality of life.

## Introduction

Peripheral arterial disease (PAD) is a prevalent condition and it results from stenosis of the pelvic and leg arteries due to atherosclerotic plaques [1]. More than 200 million people have been diagnosed with PAD globally [2]. The incidence of diabetes mellitus (DM) is on the rise worldwide and is poised to reach pandemic proportions. Studies indicate that close to half a billion people now suffer from DM globally [3]. Several predisposing factors, including DM, exacerbate PAD [4]. Diabetic patients have a more than two-fold increased prevalence of PAD compared to non-diabetics [5].

Studies have shown that PAD incidence in patients with diabetes ranges from 8 to 33% [6]. In patients with DM, PAD is also a significant predictor of lower limb amputation, often associated with other vascular events such as stroke and myocardial infarction [7]. PAD is typically asymptomatic; the presence of peripheral neuropathy, which is a frequent complication of DM, may alter pain sensitivity and bring about intermittent claudication (IC)[5]. Toe brachial index (TBI) is an effective tool to assess the risk of PAD in diabetic patients than the ankle-brachial index (ABI) because the arteries in these patients often have medial calcification, which may result in a falsely normal ABI value [8,9]. TBI is relatively immune to this phenomenon, thus constituting a potentially accurate marker of PAD as well as a predictor of general arterial atherosclerosis in diabetic patients [10].

Several studies have reported that patients with foot ulcers have a higher prevalence of PAD with consequent higher mortality and rates of lower limb amputations compared to patients without foot ulcers [11-13]. However, there have been no studies from Jordan that assess the prevalence of asymptomatic PAD among diabetic patients with foot ulcers. The prevalence of diabetic foot ulcers in the diabetic population in Jordan is 5.3% [14], and the country has many specialized diabetic foot clinics dedicated to managing the growing number of diabetic patients. This condition presents a significant challenge, particularly in the setting of asymptomatic PAD. This study aimed to determine the prevalence of asymptomatic PAD and analyze its risk factors among Jordanian diabetic patients with foot ulcers.

## Materials and methods

Study design and population

A cross-sectional study was carried out to evaluate PAD in patients with diabetic foot ulcers attending the diabetic foot clinic in the Medical City at Royal Medical Services in Amman, Jordan from January 2023 to March 2024. A total of 133 patients were included in this study. The patients were categorized into two groups: the PAD group and the non-PAD group. The PAD group was further classified into symptomatic and asymptomatic groups. Ethical approval was obtained from the institutional ethics committee (approval no: 29) in September 2022 before the study commenced. All participants provided written informed consent before participating.

Inclusion and exclusion criteria

All patients who had been diagnosed with type 2 DM, aged 18 years or above, and suffering from a diabetic foot ulcer were included. Patients who had undergone a below- or above-knee amputation, forefoot amputation, big-toe amputation, patients with an ulcer on the big toe, and those with a history of dementia or chronic obstructive pulmonary disease requiring oxygen were excluded. Moreover, patients with pseudo-claudication in spinal stenosis, nerve root compression, vasculitis, deep venous thrombosis, Raynaud’s disease, scleroderma, and those previously known to have or diagnosed with PAD according to the medical record were also excluded.

Data collection

The data were collected in the diabetic foot clinic (which has two examination rooms) by two diabetic podiatry nurses who are well-trained in TBI measurement. The medical records were used to gather data on cholesterol, triglyceride, high-density lipoprotein cholesterol (HDL-C), low-density lipoprotein cholesterol (LDL-C), and HbA1c. Data related to other risk factors of PAD were also obtained from the medical records, such as hypertension (HTN), coronary heart disease (CHD), and cerebrovascular disease (CVD). Data related to gender, age, BMI, duration of DM, and smoking status were also obtained.

Screening for PAD

The patients were instructed to not smoke, consume caffeine, or exercise within two hours of the examination. Following a 10-15-minute rest in the supine position, a vascular testing system (Smart Doppler 30EX, Hadeco Inc, Kawasaki, Japan) with photoplethysmography (PG-21) was used to measure the brachial systolic pressure (BSP) and the toe systolic pressure (TSP) bilaterally. All recordings were performed in a temperature-controlled clinical room at 23-25°C and in a dark environment while measuring toe pressure. Brachial pressure was measured by wrapping the cuff around the upper arm using an appropriate cuff width and placing a Doppler probe on the arm at a 45° to 60° angle to the skin surface. The cuff inflated until the Doppler signal obscured; then the cuff was deflated slowly, and the reading obtained was noted as BSP. The above test was also conducted on the other arm.

The toe pressure was measured by using a suitable cuff (1.5-2.5 cm) that fitted around the proximal phalanx of the big toe; the blood flow was assessed using a PPG probe, which was placed on the distal end of the big toe and attached with adhesive tape. The cuff was inflated until the waveform was obliterated, then slowly released, and the reading obtained was documented as TSP. Two values were recorded at three-minute intervals; the maximum value was used, and the test was repeated on the other foot. TBI was determined by dividing TSP by the highest reading of BSP [10].

The San Diego Claudication Questionnaire (SDCQ) - a self-administered questionnaire - was used to assess classic intermittent claudication, atypical leg symptoms, or none [15]. The tool demonstrated a sensitivity ranging from 4 to 69.9% and specificity ranging from 49.4 to 98.5% [16]. In our study, the sensitivity was found to be 65.3% while the specificity was 92.3%. For the purpose of this study, patients with TBI <0.7 and no leg symptoms per the SDCQ were considered to have asymptomatic PAD. 

Statistical analysis

Frequency and percentages were used to present categorical data, while mean and standard deviation (SD) were used for scale data. The chi-square test or Fisher's exact test, as appropriate, was used to find an association between categorical data, while an independent test was utilized to investigate mean differences of scale data based on PAD groups; binary logistic regression was used to predict factors associated with symptomatic PAD. The alpha level was set at 0.05; if the obtained p-value was less than alpha, the results were deemed statistically significant. All statistical analyses were performed using SPSS Statistics version 28.0 (IBM Corp., Armonk, NY).

## Results

A total of 133 patients participated in the study. Most were male (n=91, 68.4%) and 50 patients (37.6%) were current smokers. Hypertension was reported in 96 patients (72.2%), while 32 patients (24.1%) had CVD (Table 1).

**Table 1:** General categorical characteristics of the study sample (n=133)

Variables	Category	Frequency	Percentage
Gender	Males	91	68.4
	Females	42	31.6
Smoking status	Smoker	50	37.6
	Ex-smoker	40	30.1
	Never smoked	43	32.3
Hypertension	No	37	27.8
	Yes	96	72.2
Cerebrovascular disease	No	101	75.9
	Yes	32	24.1

The mean age of the cohort was 59.62 years (SD=12.06) while the mean BMI was 29.92 kg/m^2^ (SD=6.22). The average duration of DM was 17.77 years (SD=8.36). Regarding lipids profiles, the average HDL level was 40.32 mg/dl (SD=12.32) while the average LDL was 109.91 mg/dl (SD=42.52 mg/dl). The mean cholesterol level was 172.39 mg/dl (SD=51.28) (Table 2).

**Table 2:** General quantitative characteristics of the study sample (n=133) BMI: body mass index; HDL: high-density lipoprotein; LDL: low-density lipoprotein; SD: standard deviation

Variables	Mean	SD
Age, years	59.62	12.06
BMI, kg/m^2^	29.92	6.22
Duration of diabetes mellitus, years	17.77	8.36
HDL, mg/dl	40.32	12.32
LDL, mg/dl	109.91	42.52
Cholesterol, mg/dl	172.39	51.28

As per the operational criteria for determining PAD, 38 (28.6%) patients suffered from the condition while 95 patients (71.4%) did not. Regarding factors associated with PAD, the male gender exhibited a significantly higher proportion of PAD than the females (81.6% vs. 18.4%, p=0.039). PAD was reported in 47.4% of ex-smoker patients compared to 39.5% and 13.2% of smokers and never- smokers respectively (p=0.003). Similarly, CVD was reported in 53.1% of patients with PAD vs. 20.8% of patients without (p=0.001). while hypertension status was not significantly correlated with PAD (Table 3).

**Table 3:** Categorical factors associated with PAD PAD: peripheral arterial disease

Variables	Category	PAD		Test value	P-value
		No: 95 (71.4%)	Yes: 38 (28.6%)		
		N (%)	N (%)		
Gender	Males	60 (63.2)	31 (81.6)	4.263	0.039
	Females	35 (36.8)	7 (18.4)		
Smoking status	Smoker	35 (36.8)	15 (39.5)	11.389	0.003
	Ex-smoker	22 (23.2)	18 (47.4)		
	Never smoked	38 (40.0)	5 (13.2)		
Hypertension	No	25 (26.3)	12 (31.6)	0.374	0.541
	Yes	70 (73.7)	26 (68.4)		
Cerebrovascular disease	No	80 (79.2)	21 (20.8)	12.448	0.001
	Yes	15 (46.9)	17 (53.1)		

Furthermore, patients with PAD had a significantly higher mean age [64.42 years (SD=10.63)] compared to those without PAD [57.71 years (SD=12.11) p=0.003]. The average duration of DM was also significantly higher among those with PAD [21.03 years (SD=8.51)] compared to those without it [16.47 years (SD=7.98) p=0.004]. Conversely, the mean HDL level was significantly lower among patients with PAD [36.56 mg/dl, (SD=12.20)] compared to those without PAD [41.83 mg/dl, SD=12.11 p=0.025]. On the other hand, BMI, LDL, and cholesterol levels showed no significant differences between the two groups (p>0.05) (Table 4).

**Table 4:** Differences in quantitative variables between PAD groups BMI: body mass index; HDL: high-density lipoprotein; LDL: low-density lipoprotein; PAD: peripheral arterial disease; SD: standard deviation

Variables	PAD				Test value	P-value
	No: 95 (71.4%)		Yes: 38 (28.6%)			
Age, years	Mean	SD	Mean	SD	2.987	0.003
	57.71	12.11	64.42	10.63		
BMI, kg/m^2^	29.74	5.06	30.38	8.53	0.540	0.590
Duration of diabetes mellitus, years	16.47	7.98	21.03	8.51	2.916	0.004
HDL, mg/dl	41.83	12.11	36.56	12.20	2.265	0.025
LDL, mg/dl	108.08	37.93	114.48	52.58	0.783	0.435
Cholesterol, mg/dl	172.31	49.82	172.58	55.45	0.027	0.978

After adjusting for patients’ gender, age, smoking status, duration of DM, CVD, and HDL levels were significantly associated with PAD. The results of multiple binary logistic regression (Table 5) showed that the overall model accuracy was 77.4% (with R^2^=30.6% of the variation in PAD explained by the factors collectively). Among the predictors, only two factors showed a significant impact on the development of PAD; ex-smoker patients were 4.75 times [95% confidence interval (CI): 1.26-17.90, p=0.021) more likely to have PAD than non-smoker patients, and the odds ratio (OR) of having PAD increased by 1.05 times for each additional year of patient age (95% CI: 1.004-1.095, p=0.032)

**Table 5:** Multiple binary logistic test for predicting PAD CI: confidence interval; OR: odds ratio; PAD: peripheral arterial disease; SE: standard error

Predictors	B	SE	Wald	P-value	OR	95% CI for OR	
						Lower	Upper
Gender (male)	0.324	0.577	0.316	0.574	1.383	0.447	4.285
Age	0.047	0.022	4.623	0.032	1.049	1.004	1.095
Smoker	1.271	0.658	3.732	0.053	3.566	0.982	12.954
Ex-smoker	1.559	0.677	5.307	0.021	4.753	1.262	17.901
Duration of diabetes mellitus	0.046	0.029	2.487	0.115	1.047	0.989	1.108
Cerebrovascular disease (yes)	0.710	0.502	2.002	0.157	2.033	0.761	5.434
HDL (yes)	-0.021	0.021	0.983	0.322	0.979	0.939	1.021
Constant	-5.322	1.701	9.786	0.002	0.005		

As mentioned previously, PAD was seen in 38 out of 133 patients (28.6%). Among those, 26 patients (68.4%) were asymptomatic compared to 12 patients (31.6%) who experienced leg symptoms. In investigating factors associated with asymptomatic PAD, only hypertension showed significant results: (80.8%) of patients with hypertension had PAD compared to 19.2% of patients without (p=0.016), as shown in Tables 6-7.

**Table 6:** Categorical factors associated with symptomatic PAD ^F^Fisher's exact test; ^X^Chi-square test PAD: peripheral arterial disease

Variables	Category	PAD		Test value	P-value
		Symptomatic: 12 (31.6%)	Asymptomatic: 26 (68.4%)		
		N (%)	N (%)		
Gender	Males	11 (91.7)	20 (76.9)	1.188^F^	0.395
	Females	1 (8.3)	6 (23.1)		
Smoking status	Smoker	8 (66.7)	7 (26.9)	5.448^F^	0.066
	Ex-smoker	3 (25.0)	15 (57.7)		
	Never smoked	1 (8.3)	4 (15.4)		
Hypertension	No	7 (58.3)	5 (19.2)	5.810^X^	0.016
	Yes	5 (41.7)	21 (80.8)		
Cerebrovascular disease	No	7 (33.3)	14 (66.7)	0.067^X^	0.796
	Yes	5 (29.4)	12 (70.6)		

**Table 7:** Differences in quantitative variables between symptomatic and asymptomatic PAD groups BMI: body mass index; HDL: high-density lipoprotein; LDL: low-density lipoprotein; PAD: peripheral arterial disease; SD: standard deviation

Variables	PAD				Test value	P-value
	Symptomatic: 12 (31.6%)		Asymptomatic: 26 (68.4%)			
Age, years	Mean	SD	Mean	SD	0.620	0.539
	62.83	9.11	65.15	11.53		
BMI, kg/m^2^	28.51	6.05	31.27	9.49	0.918	0.365
Duration of diabetes mellitus, years	18.17	8.92	22.35	8.15	1.426	0.162
HDL, mg/dl	34.98	15.86	37.28	10.38	0.535	0.596
LDL, mg/dl	94.42	59.33	123.73	47.54	0.1.633	0.111
Cholesterol, mg/dl	162.50	57.87	177.23	54.84	0.757	0.454

## Discussion

Among diabetic foot patients with a TBI <0.7, 28.6% were found to have PAD, which aligns with the findings of Moosa et al. [17], in which PAD was diagnosed in 29% of diabetic patients (TBI <0.7). Their study was the only one involving diabetic patients in Jordan using TBI and it endorses our results in this population. Prior studies have shown that 14-27% of patients referred for distal pressure measurements had a low TBI [18-20]. However, the prevalence of PAD in the current study is higher than the 20.2% rate reported by Nasser et al. [21] in Bahrain based on ABI measurement. The difference may be due to the exclusion of patients with an ABI ≥1.4 in their study, based on the assumption of non-compressible arteries, although these patients could have PAD. Our results in terms of prevalence are also higher than the 17% rate in type 2 DM patients based on ABI in a study by Sartore et al. [22]. However, the prevalence of PAD in the current study is lower than the 41% rate based on TBI measurement in a study by Darban et al. [10]. This could be due to differences in participants’ age, the regime’s strictness, or control over diabetic status.

In this study, male gender, ex-smoker status, CVD, age, duration of DM, and HDL were significantly associated with PAD as evidenced by the Chi-square test. In multiple binary logistic regression, the aforementioned variables were accounted for by R²=30.6% of the variation in PAD. However, only ex-smoking and age showed a significant impact on the development of PAD. Ex-smokers were 4.75 times (95% CI: 1.26-17.9, p=0.021) more likely to have PAD than non-smokers. The Rotterdam Study by Meijer et al. [23] showed that PAD constituted a 1.15x greater risk among former smokers than never-smokers. Also, in the Health Professionals Follow-up Study (HPFS), smoking was found to be associated with a significant increase in incident clinical PAD, even in patients who had quit smoking 20 years ago [24]. Each one-year increase in age was associated with a 1.05 times risk of having PAD. There is growing evidence that PAD risk increases with age and affects a major proportion of the older population [4].

In this study, the prevalence of asymptomatic PAD was defined as a TBI <0.7 and 19.6%; asymptomatic PAD was seen in 19.6% of the diabetic foot patients while symptomatic PAD prevalence was 9%. Many studies have reported that asymptomatic PAD is several times more common than symptomatic types [4,25,26], which could be attributed to the presence of neuropathy in some patients, as it can impair pain perception and mask the symptoms of PAD. The absence of symptoms in asymptomatic PAD could also be explained by the fact that some patients may reduce their walking speed to prevent leg discomfort during exertion [26]. While examining the factors associated with asymptomatic PAD in this study, only hypertension showed significant results: 80.8% of patients with hypertension had PAD compared to 19.2% of patients without (p=0.016). Several studies in the literature have endorsed this finding, as they have reported that hypertension is one of the most significant risk factors for PAD [27-29].

Strengths of the study

This study is the first of its kind in Jordan addressing asymptomatic PAD by using TBI and SDCQ. Moreover, the diabetic foot clinic in Medical City at the Royal Medical Services receives patients from all over the country, making our study population representative of the broader Jordanian population.

Limitations of the study

The major limitation of this study is that all PAD participants were identified by TBI, which is a noninvasive vascular laboratory measurement and not a gold standard test for diagnosing PAD. Furthermore, no reference test was employed to prove the actual presence of PAD in those patients.

Recommendations

Diabetic foot patients should be screened for asymptomatic PAD at the beginning of their treatment course since the presence of PAD would delay wound healing and may lead to limp loss and poorer quality of life. In addition, we recommend using TBI as it can provide a strong indication for lower limb ischemia in patients with diabetes when medial calcification of the arteries is frequently present, without any harm to the patients as it is a noninvasive measurement. Also, we recommend extensive further research in this vital field, preferably involving studies with large sample sizes, to gain deeper insights into the correlation between patients with diabetic foot ulcers and asymptomatic PAD.

## Conclusions

The prevalence of asymptomatic PAD was 68.4% among PAD patients in this study, and it was found in 19.6% of diabetic foot patients. Hypertension was significantly associated with asymptomatic PAD. Many healthcare professionals may initiate local ulcer treatment without adequately assessing vascular status, especially in diabetic patients without intermittent claudication. Such an oversight can delay the healing process in an already complex treatment setting. TBI is a simple, noninvasive test that can effectively assess PAD in diabetic patients who may have medial calcification. Hence, diabetic patients with ulcers should be screened for PAD by using TBI, even if they have no symptoms of claudication, to establish an appropriate treatment plan.
